# A deterministic model for one-dimensional excluded flow with local interactions

**DOI:** 10.1371/journal.pone.0182074

**Published:** 2017-08-10

**Authors:** Yoram Zarai, Michael Margaliot, Anatoly B. Kolomeisky

**Affiliations:** 1 School of Electrical Engineering, Tel-Aviv University, Tel-Aviv 69978, Israel; 2 School of Electrical Engineering and the Sagol School of Neuroscience, Tel-Aviv University, Tel-Aviv 69978, Israel; 3 Department of Chemistry, Rice University, Houston, TX 77005-1892, United States of America; Universitatsklinikum Wurzburg, GERMANY

## Abstract

Natural phenomena frequently involve a very large number of interacting molecules moving in confined regions of space. Cellular transport by motor proteins is an example of such collective behavior. We derive a deterministic compartmental model for the unidirectional flow of particles along a one-dimensional lattice of sites with nearest-neighbor interactions between the particles. The flow between consecutive sites is governed by a “soft” simple exclusion principle and by attracting or repelling forces between neighboring particles. Using tools from contraction theory, we prove that the model admits a unique steady-state and that every trajectory converges to this steady-state. Analysis and simulations of the effect of the attracting and repelling forces on this steady-state highlight the crucial role that these forces may play in increasing the steady-state flow, and reveal that this increase stems from the alleviation of traffic jams along the lattice. Our theoretical analysis clarifies microscopic aspects of complex multi-particle dynamic processes.

## Introduction

Biological processes are governed by complex interactions between multiple particles that are confined in special compartments [1]. One of the most important examples of such processes is biological intracellular transport, which is carried by motor proteins (e.g., kinesins, dyneins, and myosins) [2]. These motor proteins, which are also known as biological molecular motors, can catalyze the reaction of adenosine triphosphate (ATP) hydrolysis, while at the same time converting the energy produced during this chemical reaction into a mechanical work required for their movements along cellular filaments (such as microtubules and actin filaments) [2].

Experimental observations clearly show that motor proteins usually function in large groups, suggesting that the interactions between the motors cannot be ignored [3, 4]. Understanding the collective behavior of molecular motors is critical for uncovering mechanisms of complex biological processes [2, 5, 6]. From a theoretical point of view, intracellular transport processes are usually described using non-equilibrium multi-particle lattice models [3]. In these models, the molecular motors are typically represented by particles that hop along the lattice, and the lattice sites model the binding locations of the motors along the filaments (or tracks). For a general review on transport and traffic phenomena in biological systems see for example [2–5].

A standard model from non-equilibrium statistical mechanics for molecular motors traffic (and numerous other processes) is the *totally asymmetric simple exclusion process* (TASEP) [7–9]. This is also the standard model for ribosome flow during mRNA translation (see, e.g. [8, 10, 11]). In TASEP, particles hop randomly along a unidirectionally ordered lattice of sites. Simple exclusion means that a particle cannot move into a site that is already occupied by another particle, and thus each site can be either empty or occupied by a single particle. This models moving biological particles like ribosomes and motor proteins that have volume and thus cannot overtake a moving particle in front of them. This hard exclusion principle creates an intricate indirect coupling between the particles. In particular, a slowly moving particle may lead to the formation of a traffic jam behind it.

To describe moving biological molecules with large sizes, a version of *TASEP with extended objects* has been introduced and analyzed [12–14]. In this model, each particle covers *ℓ* > 1 lattice sites. Thus a particle occupies sites *i*, …, *i* + *ℓ* − 1 for some *i*, and it can hop to site *i* + 1 provided that site *i* + *ℓ* is empty. This is used, for example, for modeling mRNA translation as it is known that every ribosome (the particle) covers several codons (sites) along the mRNA molecule [13].

There exist two versions of TASEP that differ by their boundary conditions. In TASEP with *open boundary conditions* the two sides of the chain are connected to two particle reservoirs with constant concentrations, and the particles can hop into the lattice chain (if the first site is empty) and out of the chain (if the last site is occupied). In the open boundary *homogeneous* TASEP (HTASEP), all the transition rates within the lattice are assumed to be equal and normalized to one, and thus the model is specified by an input rate *α*, an exit rate *β*, and a parameter *N* denoting the number of sites along the lattice. In TASEP with *periodic boundary conditions* the chain is closed into a ring, and a particle that hops from the last site returns to the first site. TASEP has been widely utilized for studying various natural and artificial processes, including vehicular traffic flow, mRNA translation, surface growth, communication networks, and more [3, 15].

Ref. [16] used HTASEP with periodic boundary conditions to analyze transport on a lattice in the presence of local interactions between particles and substrate, illustrating the effect of local conformation of the substrate on the characteristics of the flow of molecular motors. TASEP with particle interactions and with periodic boundary conditions was studied in [17], and with open boundary conditions in [18–21]. Specifically, the authors in [20, 21] proposed a modified TASEP model that incorporates the realistic observed feature of nearest-neighbor interactions. In this model, the transition rate in every site along the lattice depends on the states of four consecutive sites. Their conclusions were that weak repulsive interaction results in maximal flux, and that the molecular motors are influenced more strongly by attractive interactions.

Unfortunately, rigorous analysis of TASEP is non-trivial, and exact solutions exist only in special cases, for example when considering the model with the *homogeneous* rates (HTASEP). Typically, the non-homogeneous case and cases that include other local interactions are only studied via various approximations and extensive Monte Carlo computer simulations. These simulations are run until convergence to a (stochastic) steady-state, yet without a rigorous proof that convergence indeed takes place for all the feasible parameter values.

In this paper, we introduce a new *deterministic* model for the flow of motor proteins along a one-dimensional lattice of sites with nearest-neighbor interactions between the motors. The flow of the motor proteins is unidirectional, and it satisfies a “soft” simple exclusion principle. The nearest-neighbor effect is modeled by two “force” interactions with parameters *q* and *r*. It is more convenient to explain the effect of these interactions in “particle-like” terms, although in the new model the density in every site takes values in the range [0, 1] (and not {0, 1}).

Consider a transition of a particle from site *i* to site *i* + 1. If site *i* + 2 is already occupied then the rate of movement depends on a parameter *q* ≥ 0 that represents an “attachment/detachment force” when generating *new* neighbors. A value *q* > 1 [*q* < 1] means that the particle will tend [not] to hop forward, as there is a strong attraction [repulsion] to the particle in site *i* + 2. On the other-hand, if site *i* − 1 is already occupied then the rate of movement depends on a parameter *r* ≥ 0 that represents an “attachment/detachment force” when breaking from *old* neighbors. A value *r* > 1 [*r* < 1] means that the particle will tend [not] to hop forward, as there is a strong repulsion [attraction] from the neighboring particle in site *i* − 1. A value of *q* = 1 [*r* = 1] implies no attachment/detachment force when generating new neighbors [when breaking from old neighbors].

An important advantage of our model is that it is highly amenable to rigorous analysis even for *non-homogenous* transition rates. We prove, for example, that the dynamics always converges to a steady-state density along the lattice. Thus, the flow also converges to a steady-state value. This steady-state depends on the lattice size, the transition rates, and the parameters *q*, *r*, but not on the initial density along the lattice (i.e. the initial conditions). Analysis and simulations of the effect of the attracting and repelling forces on this steady-state highlight the crucial role that these forces may play in increasing the steady-state flow, and reveal that this increase stems from the alleviation of traffic jams along the lattice. It is well-known that molecular motors indeed form traffic jams and that these have important biological implications (see, e.g. [22–24]). In particular, analysis and simulations of the model reveal a new regime that may be interpreted as the “opposite” of a traffic jam along the lattice.

Our approach extends a deterministic mathematical model that has been used for describing and analyzing the flow of ribosomes along the mRNA molecule during the process of mRNA translation. The next section provides a brief overview of this model.

### The Ribosome Flow Model (RFM)

**Fig 1:**
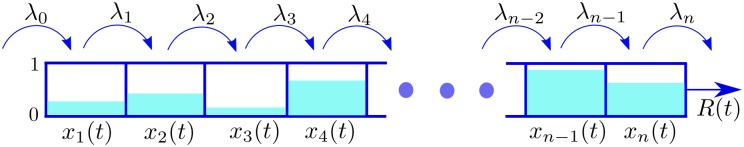
The RFM models unidirectional flow along a chain of *n* sites. The state variable *x*_*i*_(*t*) ∈ [0, 1] represents the density at site *i* at time *t*. The parameter *λ*_*i*_ > 0 controls the transition rate from site *i* to site *i* + 1, with *λ*_0_ > 0 [*λ*_*n*_ > 0] controlling the initiation [exit] rate. The output rate at time *t* is *R*(*t*) ≔ *λ*_*n*_ *x*_*n*_(*t*).

The RFM [25] is a nonlinear, continuous-time, compartmental model for the unidirectional flow of “material” along a one-dimensional chain of *n* consecutive compartments. It can be derived via a mean-field approximation of TASEP with open boundary conditions [3, Section 4.9.7] [7, p. R345]. The RFM includes *n* + 1 parameters: *λ*_0_ > 0 controls the initiation rate, *λ*_*n*_ > 0 the exit rate, and *λ*_*i*_ > 0, *i* = 1, …, *n* − 1, the transition rate from site *i* to site *i* + 1. The state variable $x_{i}\left(t\right):R_{+}\to \left[0,1\right]$, *i* = 1, …, *n*, describes the normalized amount of “material” (or density) at site *i* at time *t*, where *x*_*i*_(*t*) = 1 [*x*_*i*_(*t*) = 0] indicates that site *i* is completely full [completely empty] at time *t*. Thus, the vector $x\left(t\right)≔{\left[\begin{array}{ccc} x_{1}\left(t\right) & \ldots & x_{n}\left(t\right) \end{array}\right]}^{\prime }$ describes the density profile along the chain at time *t*. The output rate at time *t* is *R*(*t*) ≔ *λ*_*n*_ *x*_*n*_(*t*) (see Fig 1).

Let *x*_0_(*t*) ≡ 1, and *x*_*n*+1_(*t*) ≡ 0. The dynamics of the RFM with *n* sites is given by the following set of *n* nonlinear ODEs:

$$\begin{array}{c} {\dot{x}}_{i}=\lambda_{i-1}x_{i-1}\left(1-x_{i}\right)-\lambda_{i}x_{i}\left(1-x_{i+1}\right),\;i=1,\ldots ,n. \end{array}$$ (1)

This can be explained as follows. The flow of material from site *i* to site *i* + 1 at time *t* is *λ*_*i*_ *x*_*i*_(*t*)(1 − *x*_*i*+1_(*t*)). This flow increases with the density at site *i*, and decreases as site *i* + 1 becomes fuller. This corresponds to a “soft” version of a simple exclusion principle. Note that the maximal possible flow from site *i* to site *i* + 1 is the transition rate *λ*_*i*_. Thus Eq (1) simply states that the change in the density at site *i* at time *t* is the input rate to site *i* (from site *i* − 1) at time *t* minus the output rate (to site *i* + 1) at time *t*.

The trajectories of the RFM evolve on the compact and convex state-space

$$\begin{array}{c} C^{n}≔\left\{x\in R^{n}:x_{i}\in \left[0,1\right],i=1,\ldots ,n\right\}. \end{array}$$

Let Int(*C*^*n*^) [∂*C*^*n*^] denote the interior [boundary] of *C*^*n*^. Ref. [26] has shown that the RFM is a *tridiagonal cooperative dynamical system* [27], and consequently Eq (1) admits a *unique* steady-state density *e* = *e*(*λ*_0_, …, *λ*_*n*_) ∈ Int(*C*^*n*^) that is globally asymptotically stable, that is, lim_*t* → ∞_ *x*(*t*, *a*) = *e* for all *a* ∈ *C*^*n*^ (see also [28]). This means that trajectories corresponding to different initial conditions all converge to the same steady-state density *e*. In particular, the density at the last site *x*_*n*_(*t*) converges to the value *e*_*n*_, so the output rate *R*(*t*) converges to a steady-state value *R* ≔ *λ*_*n*_ *e*_*n*_.

An important advantage of the RFM (e.g. as compared to TASEP) is that it is amenable to mathematical analysis using tools from systems and control theory. Furthermore, most of the analysis hold for the general, non-homogeneous case (i.e. the case where the transition rates *λ*_*i*_ differ from one another). For more on the analysis of the RFM and its biological implications, see [26, 28–37].

In this paper, we extend the RFM to include nearest-neighbor interactions, namely, binding and repelling actions that are dynamically activated for each site based on the state of its neighboring sites. A parameter *r* [*q*] controls the binding/repelling forces between two existing [new] neighbors. We refer to the new model as the *excluded flow with local repelling and binding model* (EFRBM). It is important to note that this is significantly different from the RFM. For example, the EFRBM, unlike the RFM, is *not* a cooperative system [27]. Also, in the RFM the dynamics at site *i* is directly affected by its two nearest neighbors sites, whereas in the EFRBM the dynamics is directly affected by the density in four neighboring sites. Thus, unlike the RFM, the EFRBM is not a tridiagonal system. Also, the RFM has been used to model ribosome flow, whereas here we apply the EFRBM to study the flow of motor proteins.

We show that the EFRBM is a contractive dynamical system. This holds for any set of feasible transition rates and local interaction forces including the case of non-homogeneous transition rates. This implies that the EFRBM admits a unique steady-state that is globally asymptotically stable. Thus, every set of parameters corresponds to a unique steady-state output rate. We analyze the behavior of this steady-state under the assumption *rq* = 1 that follows from fundamental thermodynamic arguments (see [38]). We show that a small neighbor-repelling force (i.e. small *r* and thus a large *q* = 1/*r*) leads to a small output rate. Analysis and simulations show that this is due to the formation of traffic jams at the beginning of the lattice. On the other-hand, a strong neighbor-repelling force (i.e. large *r* and small *q*) lead to a high output rate. In this case, an interesting phenomena emerges: the density in every second site goes to zero. This “separation of densities” is the “opposite” of a traffic jam. These results highlight the impact of traffic jams on the output rate.

The remainder of this paper is organized as follows. The next section describes the EFRBM. The following two sections describe our main analysis results and their biological implications. This includes analysis of the asymptotic behavior of the EFRBM, and the effects of the nearest-neighbor interactions on the steady-state behavior of the EFRBM. The final section summarizes and describes several directions for further research. To increase the readability of this paper, all the proofs are placed in the Appendix.

## The EFRBM

The EFRBM with *n* sites includes *n* + 3 parameters:

- *λ*_*i*_ > 0, *i* = 0, …, *n*, controls the transition rate from site *i* to site *i* + 1, where *λ*_0_ [*λ*_*n*_] controls the input [output] rate.
- *r* ≥ 0 is the attachment/detachment force between any two existing (consecutive) neighbors.
- *q* ≥ 0 is the attachment/detachment force between any two new (consecutive) neighbors.

**Fig 2:**
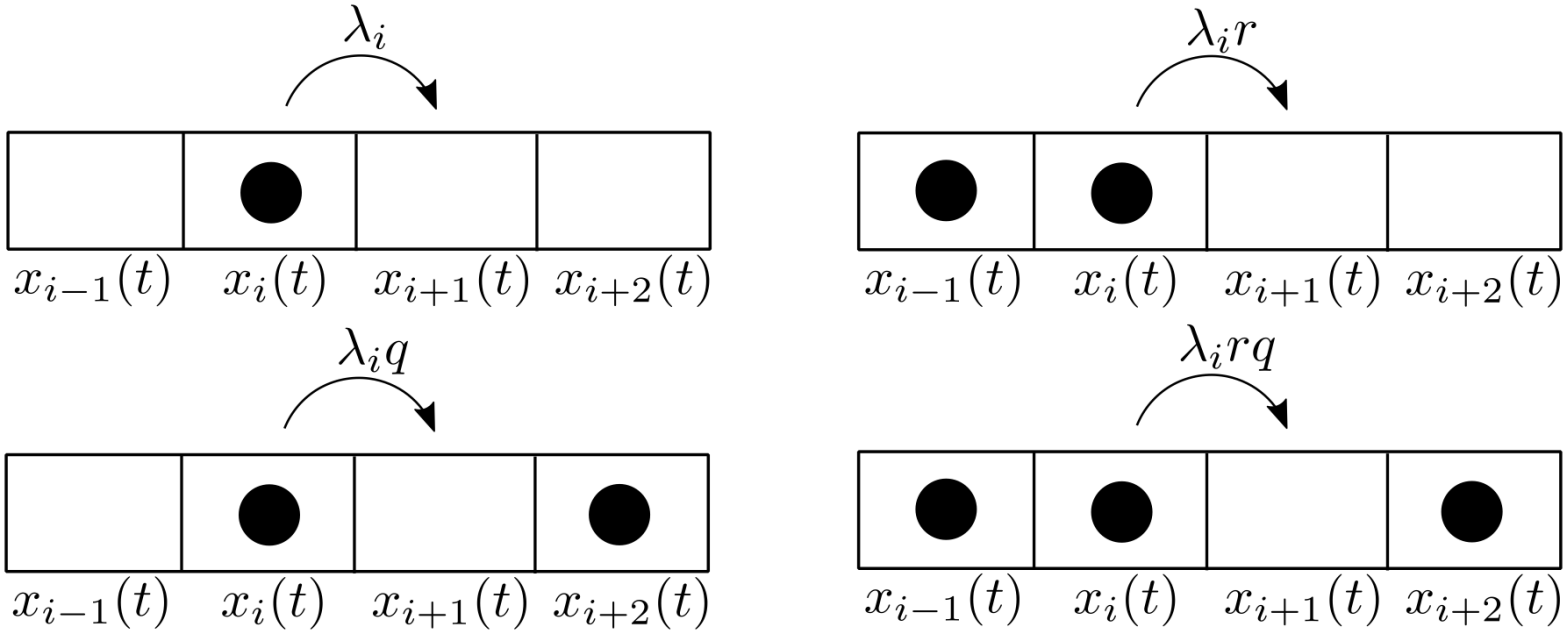
Schematic explanation of the transition flow from site *i* to site *i* + 1 in the EFRBM. Upper-left: when both sites *i* − 1 and *i* + 2 do not contain particles, the transition rate is *λ*_*i*_. Lower-left: when site *i* − 1 does not contain particles, and site *i* + 2 does, the transition rate is *λ*_*i*_ *q*. Upper-right: when site *i* − 1 contains particles, and site *i* + 2 does not, the transition rate is *λ*_*i*_ *r*. Lower-right: when both sites *i* − 1 and *i* + 2 contain particles, the transition rate is *λ*_*i*_ *rq*.

Fig 2 depicts the four possible transition scenarios from site *i* to site *i* + 1, and the rates in each case. For simplicity, we use a schematic “particle-like” explanation, although in the EFRBM the state-variables represent a normalized material density in the range [0, 1] and not a binary choice {0, 1} like in TASEP. If both sites *i* − 1 and *i* + 2 do not contain particles, the transition rate is simply *λ*_*i*_, as in the RFM. If a particle is located at site *i* − 1 [*i* + 2] but site *i* + 2 [*i* − 1] is empty then the transition rate is *λ*_*i*_ *r* [*λ*_*i*_ *q*]. If both sites contain particles the transition rate is *λ*_*i*_ *rq*.

The EFRBM also includes *n* state-variables *x*_*i*_(*t*), *i* = 1, …, *n*. Just like in the RFM, *x*_*i*_(*t*) describes the normalized density at site *i* at time *t*, where *x*_*i*_(*t*) = 0 [*x*_*i*_(*t*) = 1] means that the site is completely empty [full].

To state the dynamical equations describing the EFRBM we introduce more notation. Let *x*_0_(*t*) ≡ 1, *x*_*n*+1_(*t*) ≡ 0, and denote

$$\begin{array}{c} z_{i}\left(t\right)≔\left\{ \begin{array}{cc} x_{i}\left(t\right), & i=1,\ldots ,n,\\ 0, & \text{otherwise}. \end{array}\right. \end{array}$$ (2)

Then the EFRBM is described by

$$\begin{array}{c} {\dot{x}}_{i}=g_{i-1}\left(x\right)-g_{i}\left(x\right),\;i=1,\ldots ,n, \end{array}$$ (3)

where

$$\begin{array}{c} g_{i}\left(x\right)≔\lambda_{i}x_{i}\left(1-x_{i+1}\right)\left(1+\left(q-1\right)z_{i+2}\right)\left(1+\left(r-1\right)z_{i-1}\right). \end{array}$$ (4)

We now explain these equations. The term *g*_*i*_(*x*) represents the flow from site *i* to site *i* + 1, so Eq (3) means that the change in the density at site *i* is the inflow from site *i* − 1 minus the outflow to site *i* + 1. To explain Eq (4), consider for example the case *i* = 2 (and assume that *n* ≥ 4). Then Eq (4) yields

$$\begin{array}{c} g_{2}\left(x\right)=\lambda_{2}x_{2}\left(1-x_{3}\right)\left(1+\left(q-1\right)x_{4}\right)\left(1+\left(r-1\right)x_{1}\right). \end{array}$$ (5)

The term *x*_2_ means that the flow from site 2 to site 3 increases with the density at site 2. The term (1 − *x*_3_) represents soft exclusion: as the density at site 3 increases, the transition from site 2 to site 3 gradually decreases. The term (1 + (*q* − 1)*x*_4_) represents the fact that the flow into site 3 also depends on the density at site 4: if *q* > 1 [*q* < 1] then the transition increases [decreases] with *x*_4_, that is, the “particles” at site 4 “attract” [“repel”] the particles that move from site 2 to site 3. The term (1 + (*r* − 1)*x*_1_) is similar but represents an attachment/detachment force between the “particles” in sites 1 and 2.

Note that for *r* = *q* = 1, *g*_*i*_(*x*) = *λ*_*i*_ *x*_*i*_(1 − *x*_*i*+1_), and thus in this case the EFRBM reduces to the RFM (see Eq (1)). On the other hand, if *q* = *r* = 0 then *g*_*i*_(*x*) = *λ*_*i*_ *x*_*i*_(1 − *x*_*i*+1_)(1 − *x*_*i*+2_)(1 − *x*_*i*−1_). This represents a kind of an “extended objects” RFM, as the transition from site *i* to site *i* + 1 decreases with the density in sites *i* − 1, *i* + 1, and *i* + 2.

**Remark 1** It is useful to think of the EFRBM as an RFM with *time-varying* transition rates. For example, we can write Eq (5) as

$$\begin{array}{c} g_{2}\left(x\left(t\right)\right)=\eta_{2}\left(t\right)x_{2}\left(t\right)\left(1-x_{3}\left(t\right)\right), \end{array}$$

where *η*_2_(*t*) ≔ *λ*_2_(1 + (*q* − 1)*x*_4_(*t*))(1 + (*r* − 1)*x*_1_(*t*)). Note that this time-varying transition rate depends on *λ*_2_ (i.e., the fixed site to site transition rate), and also on *r* and *q* and the time-varying densities in the neighboring sites, as these determine the interaction forces between the moving particles.

We denote the flow from site *x*_*n*_ to the environment by

$$\begin{array}{c} R\left(t\right)≔\lambda_{n}x_{n}\left(t\right)\left(1+\left(r-1\right)x_{n-1}\left(t\right)\right). \end{array}$$ (6)

This is the *output rate* at time *t*.

**Example 1** The EFRBM with *n* = 3 sites is given by:

$$\begin{array}{ccc} {\dot{x}}_{1} & = & \lambda_{0}\left(1-x_{1}\right)\left(1+\left(q-1\right)x_{2}\right)-\lambda_{1}x_{1}\left(1-x_{2}\right)\left(1+\left(q-1\right)x_{3}\right),\\ {\dot{x}}_{2} & = & \lambda_{1}x_{1}\left(1-x_{2}\right)\left(1+\left(q-1\right)x_{3}\right)-\lambda_{2}x_{2}\left(1-x_{3}\right)\left(1+\left(r-1\right)x_{1}\right),\\ {\dot{x}}_{3} & = & \lambda_{2}x_{2}\left(1-x_{3}\right)\left(1+\left(r-1\right)x_{1}\right)-\lambda_{3}x_{3}\left(1+\left(r-1\right)x_{2}\right). \end{array}$$ (7)

If *q* = *r* = 0 then this becomes

$$\begin{array}{ccc} {\dot{x}}_{1} & = & \lambda_{0}\left(1-x_{1}\right)\left(1-x_{2}\right)-\lambda_{1}x_{1}\left(1-x_{2}\right)\left(1-x_{3}\right),\\ {\dot{x}}_{2} & = & \lambda_{1}x_{1}\left(1-x_{2}\right)\left(1-x_{3}\right)-\lambda_{2}\left(1-x_{1}\right)x_{2}\left(1-x_{3}\right),\\ {\dot{x}}_{3} & = & \lambda_{2}\left(1-x_{1}\right)x_{2}\left(1-x_{3}\right)-\lambda_{3}\left(1-x_{2}\right)x_{3}. \end{array}$$ (8)

On the other-hand, for *q* = 1 and *r* = 0 Eq (7) becomes

$$\begin{array}{ccc} {\dot{x}}_{1} & = & \lambda_{0}\left(1-x_{1}\right)-\lambda_{1}x_{1}\left(1-x_{2}\right),\\ {\dot{x}}_{2} & = & \lambda_{1}x_{1}\left(1-x_{2}\right)-\lambda_{2}\left(1-x_{1}\right)x_{2}\left(1-x_{3}\right),\\ {\dot{x}}_{3} & = & \lambda_{2}\left(1-x_{1}\right)x_{2}\left(1-x_{3}\right)-\lambda_{3}\left(1-x_{2}\right)x_{3}, \end{array}$$ (9)

and this system admits a continuum of steady-states, as ${\left[\begin{array}{ccc} 1 & 1 & s \end{array}\right]}^{\prime }$ is a steady-state for all *s*.

Following [38] (see also [39]), we view creating and breaking a pair of particles as opposite chemical transitions, so by detailed balance arguments: $\frac{q}{r}=\exp\left(\frac{E}{K_{B}T}\right)$, where *E* is the interaction energy. As in [38], we also assume that *E* is equally split between the creation and breaking processes, so

$$\begin{array}{c} q=\exp\left(\frac{E}{2K_{B}T}\right),\;r=\exp\left(\frac{-E}{2K_{B}T}\right). \end{array}$$ (10)

This has a clear physical meaning. If *E* > 0 the interaction is attractive, so the particle moves faster when creating a new pair (*q* > 1) since the energy of the system decreases by *E*. On the other-hand, breaking out of the cluster increases the energy by *E* and the transition rate is thus slowed down (*r* < 1). Similarly, the case *E* < 0 corresponds to a repulsive interaction and then *q* < 1 and *r* > 1. Note that Eq (10) implies in particular that

$$\begin{array}{c} rq=1. \end{array}$$ (11)

In this case, the EFRBM contains *n* + 2 parameters: *λ*_0_, …, *λ*_*n*_, and *r* (as *q* = 1/*r*). Note that if Eq (11) holds then Eq (4) becomes

$$\begin{array}{c} g_{i}\left(x\right)=\lambda_{i}x_{i}\left(1-x_{i+1}\right)\left(1-\frac{r-1}{r}z_{i+2}\right)\left(1+\left(r-1\right)z_{i-1}\right). \end{array}$$ (12)

The next section derives several theoretical results on the dynamical behavior of the EFRBM. Recall that all the proofs are placed in the Appendix.

## Asymptotic behavior of the EFRBM

Let *x*(*t*, *a*) denote the solution of the EFRBM at time *t* for the initial condition *x*(0) = *a* ∈ *C*^*n*^.

### Invariance and persistence

The next result shows that the *n*-dimensional unit cube *C*^*n*^ is an invariant set of the EFRBM, that is, any trajectory that emanates from an initial condition in *C*^*n*^ remains in *C*^*n*^ for all time. Furthermore, any trajectory emanating from the boundary of *C*^*n*^ “immediately enters” *C*^*n*^. This is a technical result, but it is important as in the interior of *C*^*n*^ the EFRBM admits several useful properties.

**Proposition 1** *Assume that* *q*, *r* > 0. *For any* *τ* > 0 *there exists* *d* = *d*(*τ*) ∈ (0, 1/2) *such that*

$$\begin{array}{c} d\leq x_{i}\left(t+\tau ,a\right)\leq 1-d, \end{array}$$

*for all* *a* ∈ *C*^*n*^, *all* *i* ∈ {1, …, *n*}, *and all* *t* ≥ 0.

This means that all the trajectories of the EFRBM enter and remain in the interior of *C*^*n*^ after an arbitrarily short time. In particular, both *C*^*n*^ and Int(*C*^*n*^) are invariant sets of the EFRBM dynamics.

From a biological point of view this means that if the system is initiated such that every density is in [0, 1] then this remains true for all time *t* ≥ 0, so the equations “make sense” in this respect. Furthermore, after an arbitrarily short time the densities are all in (0, 1), i.e. any completely empty [full] site immediately becomes not completely empty [full].

### Contraction

Differential analysis and in particular contraction theory proved to be a powerful tool for analyzing the asymptotic behavior of nonlinear dynamical systems. In a contractive system, trajectories that emanate from different initial conditions approach each other at an exponential rate [40–42].

For our purposes, we require a generalization of contraction with respect to (w.r.t.) a fixed norm that has been introduced in [43]. Consider the time-varying dynamical system:

$$\begin{array}{c} \dot{x}\left(t\right)=f\left(t,x\left(t\right)\right), \end{array}$$ (13)

whose trajectories evolve on an invariant set $\Omega \subset R^{n}$ that is compact and convex. Let *x*(*t*, *t*_0_, *a*) denote the solution of Eq (13) at time *t* for the initial condition *x*(*t*_0_) = *a*. The dynamical system Eq (13) is said to be *contractive after a small overshoot* (SO) [43] on *Ω* w.r.t. a norm $|\cdot |:R^{n}\to R_{+}$ if for any *ε* > 0 there exists *ℓ* = *ℓ*(*ε*)>0 such that

$$\begin{array}{c} |x\left(t,t_{0},a\right)-x\left(t,t_{0},b\right)|\leq \left(1+\varepsilon \right)\exp\left(-\left(t-t_{0}\right)\ell \right)\left|a-b\right|, \end{array}$$

for all *a*, *b* ∈ *Ω* and all *t* ≥ *t*_0_ ≥ 0. Intuitively speaking, this means that any two trajectories of the system approach each other at an exponential rate *ℓ*, but with an arbitrarily small overshoot of 1 + *ε*.

Let ${\left|\cdot \right|}_{1}:R^{n}\to R_{+}$ denote the *L*_1_ norm, i.e. for $z\in R^{n}$, |*z*|_1_ = |*z*_1_|+…+|*z*_*n*_|.

**Proposition 2** *The EFRBM with* *q*, *r* > 0 *is SO on* *C*^*n*^ *w.r.t. the* *L*_1_ *norm, that is, for any* *ε* > 0 *there exists* *ℓ* = *ℓ*(*ε*)>0 *such that*

$$\begin{array}{c} {\left|x(t,a)-x(t,b)\right|}_{1}\leq \left(1+\varepsilon \right)\exp\left(-\ell t\right){\left|a-b\right|}_{1}, \end{array}$$ (14)

*for all* *a*, *b* ∈ *C*^*n*^ *and all* *t* ≥ 0.

From a biological point of view this means the following. The state of the system at any time *t* is a vector describing the density at each site at time *t*. We measure the distance between any two density vectors using the *L*_1_ vector norm. Suppose that we initiate the system with two different densities. This generates two different solutions of the dynamical system. The distance between these solutions decreases with time at an exponential rate.

The next example demonstrates this contraction property. Let 1_*n*_ [0_*n*_] denote the column vector of *n* ones [zeros].

**Fig 3:**
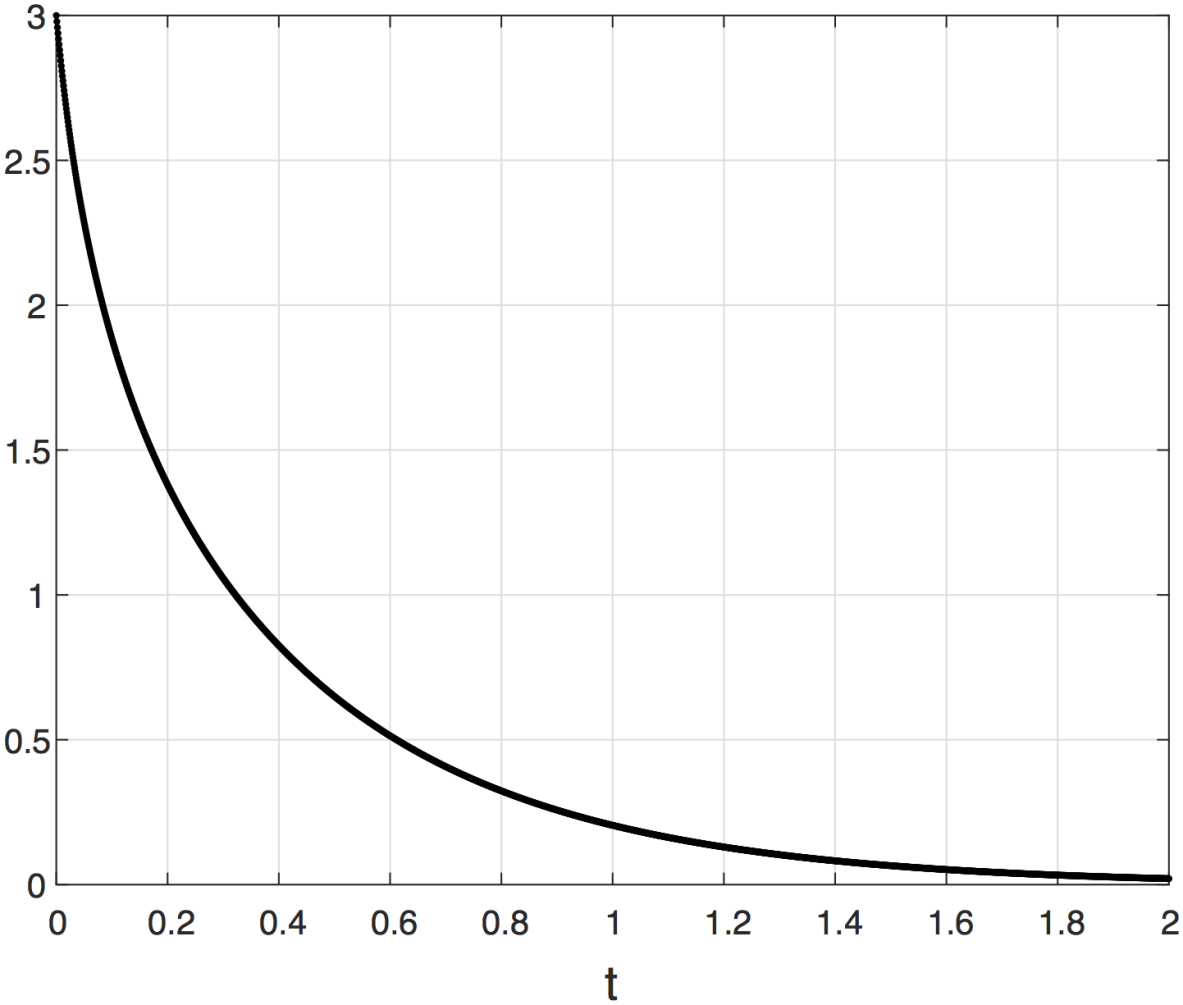
The distance |*x*(*t*, *a*) − *x*(*t*, *b*)|_1_ as a function of time for the EFRBM in Example 2.

**Example 2** Consider the EFRBM with dimension *n* = 3, and parameters *λ*_0_ = 1, *λ*_1_ = 2, *λ*_2_ = 3, *λ*_3_ = 4, *r* = 5, and *q* = 1/5. Fig 3 depicts |*x*(*t*, *a*) − *x*(*t*, *b*)|_1_, with *a* = 0_3_ and *b* = 1_3_, as a function of time for *t* ∈ [0, 2]. It may be seen that the *L*_1_ distance between the two trajectories goes to zero at an exponential rate.

Prop. 2 implies that the EFRBM satisfies several important asymptotic properties. These are described in the following subsections.

### Global asymptotic stability

Write the EFRBM Eq (3) as $\dot{x}=f\left(x\right)$. Since the compact and convex set *C*^*n*^ is an invariant set of the dynamics, it contains at least one steady-state. That is, there exists *e* = *e*(*λ*_0_, …, *λ*_*n*_, *q*, *r*) such that *f*(*e*) = 0_*n*_. By Proposition 1, *e* ∈ Int(*C*^*n*^). Using Eq (14) with *b* ≔ *e* yields the following result.

**Corollary 1** *Assume that* *q*, *r* > 0. *Then the EFRBM admits a unique steady-state* *e* ∈ Int(*C*^*n*^) *that is globally asymptotically stable, i.e.*

$$\begin{array}{c} \lim_{t\to \infty }x\left(t,a\right)=e,\;\text{for}\;\text{all}\;a\in C^{n}. \end{array}$$

This means that any solution of the EFRBM converges to a unique steady-state density (and thus a unique steady-state output rate) that depends on the rates *λ*_*i*_, and the parameters *r* and *q*, but not on the initial condition. From a biological point of view, this means that the system always converges to a steady-state density and a corresponding steady-state output rate, and thus it makes sense to study how these depend on the various parameters.

Note that the assumption that *r*, *q* > 0 cannot be dropped. Indeed, Eq (9), corresponding to a EFRBM with *n* = 3, *q* = 1 and *r* = 0, admits a continuum of steady-states.

**Fig 4:**
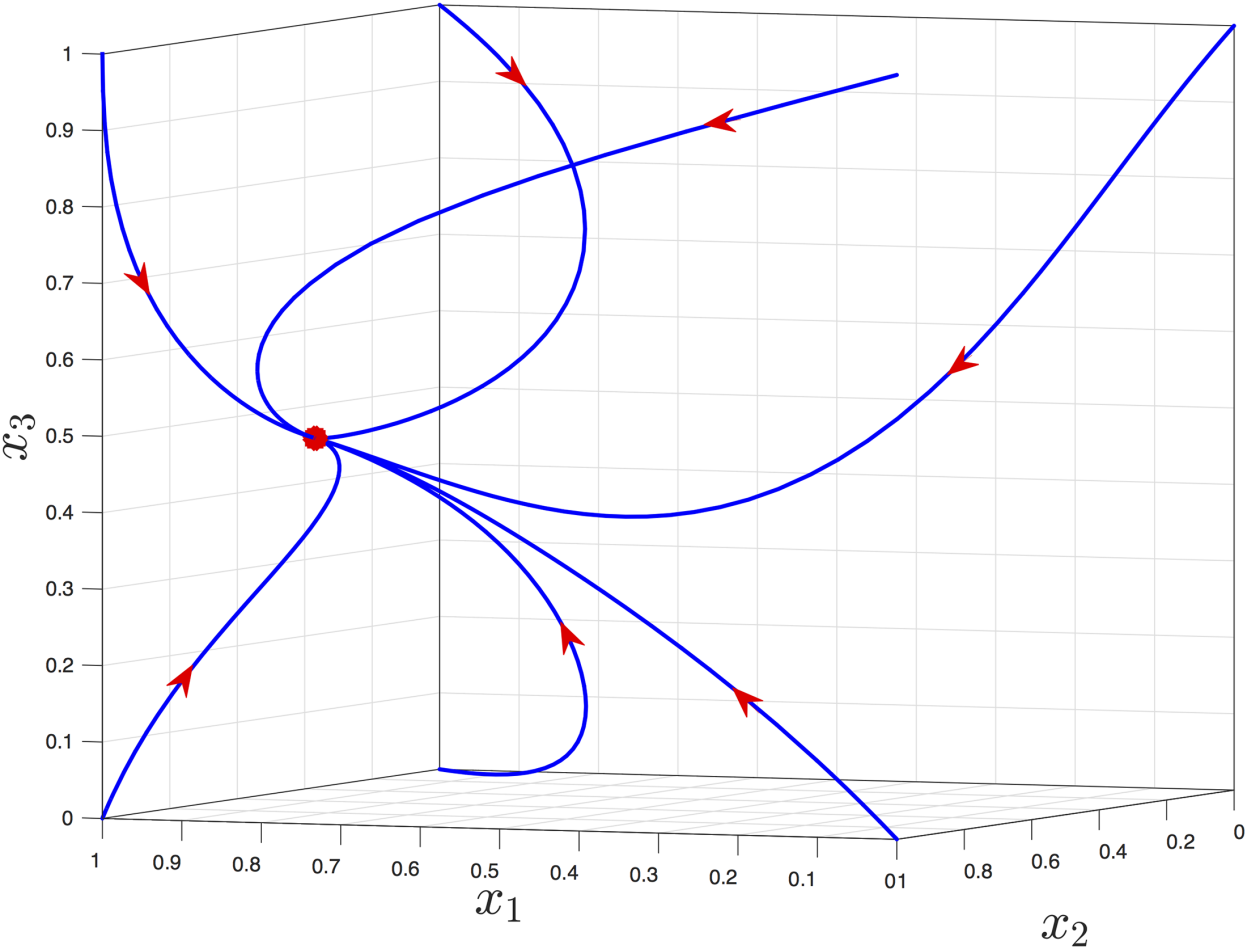
Trajectories of the EFRBM in Example 3 for seven arbitrary initial conditions. The steady-state *e* is denoted by an asterisk.

**Example 3** Fig 4 depicts the trajectories of Eq (3) with *n* = 3, *λ*_0_ = 0.5, *λ*_1_ = 0.8, *λ*_2_ = 0.7, *λ*_3_ = 0.6, *r* = 1/2, and *q* = 2, for several initial conditions. It may be seen that all trajectories converge to a unique steady-state $e={\left[\begin{array}{ccc} 0.8555 & 0.7881 & 0.4268 \end{array}\right]}^{\prime }$. (All the numerical values in the simulations described in this paper are to four digit accuracy.)

The rigorous proof that every trajectory converges to a steady-state is important, as it implies that after some time the densities are very close to their steady-state values. The next step is to analyze this steady-state density and the corresponding steady-state output rate, and explore how these are related to the various parameters of the model.

### Analysis of the steady-state

At steady-state, (i.e. for *x* = *e*) the left-hand side of all the equations in Eq (3) is zero (i.e. ${\dot{x}}_{i}=0$, *i* = 1, …, *n*), so *g*_*i*−1_(*e*) = *g*_*i*_(*e*) for all *i*. This implies that

$$\begin{array}{ccc} & & \lambda_{0}\left(1-e_{1}\right)\left(1+\left(q-1\right)e_{2}\right)\\ & & \;=\lambda_{1}e_{1}\left(1-e_{2}\right)\left(1+\left(q-1\right)e_{3}\right)\\ & & \;=\lambda_{2}e_{2}\left(1-e_{3}\right)\left(1+\left(q-1\right)e_{4}\right)\left(1+\left(r-1\right)e_{1}\right)\\ & & \;=\lambda_{3}e_{3}\left(1-e_{4}\right)\left(1+\left(q-1\right)e_{5}\right)\left(1+\left(r-1\right)e_{2}\right)\\ & & \;\vdots \\ & & \;=\lambda_{n-1}e_{n-1}\left(1-e_{n}\right)\left(1+\left(r-1\right)e_{n-2}\right)\\ & & \;=\lambda_{n}e_{n}\left(1+\left(r-1\right)e_{n-1}\right), \end{array}$$ (15)

and also that the steady-state flow satisfies

$$\begin{array}{c} R=g_{i}\left(e\right),\;i=0,\ldots ,n. \end{array}$$ (16)

In particular, *R* = *λ*_*n*_ *e*_*n*_(1 + (*r* − 1)*e*_*n*−1_) and since every *e*_*i*_ ∈ (0, 1), the steady-state flow is positive (i.e. a left-to-right flow) for any *r* > 0.

Also, for the case *rq* = 1 it follows from *R* = *λ*_0_(1 − *e*_1_)(1 + (*q* − 1)*e*_2_) that for *r* ≥ 1, *R* ≤ *λ*_0_, whereas for *r* < 1 it follows from *R* = *λ*_*n*_ *e*_*n*_(1 + (*r* − 1)*e*_*n*−1_) that *R* ≤ *λ*_*n*_, so

$$\begin{array}{c} R\leq \max\{\lambda_{0},\lambda_{n}\}. \end{array}$$

This means in particular that the output rate is always bounded.

**Fact 1** *It follows from* Eq (15) *that if we multiply all the* *λ*_*i*_s *by a parameter* *c* > 0 *then* *e* *will not change, i.e.* *e*(*cλ*) = *e*(*λ*). *Thus, by* Eq (16) *R*(*cλ*) = *cR*(*λ*), *for all* *c* > 0, *that is, the steady-state flow [density] is* homogeneous of degree one [zero] *w.r.t. the* *λ*_*i*_s.

In the spacial case *n* = 2 the steady-state equations Eq (15) can be solved in closed-form.

**Fact 2** *Consider the EFRBM with* *n* = 2 *and* *q* = 1/*r*. *Define*

$$\begin{array}{ccc} a_{1} & ≔ & \left(1-\frac{1}{r}\right)\left(\lambda_{2}r+\lambda_{1}\right)+\frac{\lambda_{1}\lambda_{2}}{\lambda_{0}}. \end{array}$$ (17)

*Then* $e={\left[\begin{array}{cc} e_{1} & e_{2} \end{array}\right]}^{\prime }$ *is given by*

$$\begin{array}{ccc} e_{2} & = & \frac{\lambda_{1}+\lambda_{2}+a_{1}-\sqrt{{\left(\lambda_{1}+\lambda_{2}+a_{1}\right)}^{2}-4a_{1}\lambda_{1}}}{2a_{1}},\\ e_{1} & = & \frac{\lambda_{2}e_{2}}{\lambda_{1}+\left(\lambda_{2}\left(1-r\right)-\lambda_{1}\right)e_{2}}. \end{array}$$ (18)

*Note that even in this case the expression for* *e* *is non-trivial*.

Let $R_{++}^{n}$ denote the set of *n* dimensional vectors with all entries positive. Let $v≔{\left[\begin{array}{ccccc} \lambda_{0} & \ldots & \lambda_{n} & r & q \end{array}\right]}^{\prime }$ denote the set of parameters in the EFRBM with dimension *n*. The results above imply that there exists a function $h:R_{++}^{n+3}\to \text{Int}\left(C^{n}\right)$ such that *e* = *h*(*v*) is the unique steady-state of the EFRBM with parameters *v*.

**Proposition 3** *The function* $h:R_{++}^{n+3}\to \text{Int}\left(C^{n}\right)$ *is analytic*.

This result allows in particular to consider the derivatives of the steady-state density *e* = *e*(*v*) and the steady-state output rate *R* = *R*(*v*) w.r.t. small changes in some of the parameters *v*, that is, the sensitivity of the steady-state w.r.t. small changes in the parameters.

## Effect of nearest-neighbor interactions

We begin with several simulations demonstrating the effect of the parameter *r* (and *q* = 1/*r*) on the steady-state of the EFRBM.

**Fig 5:**
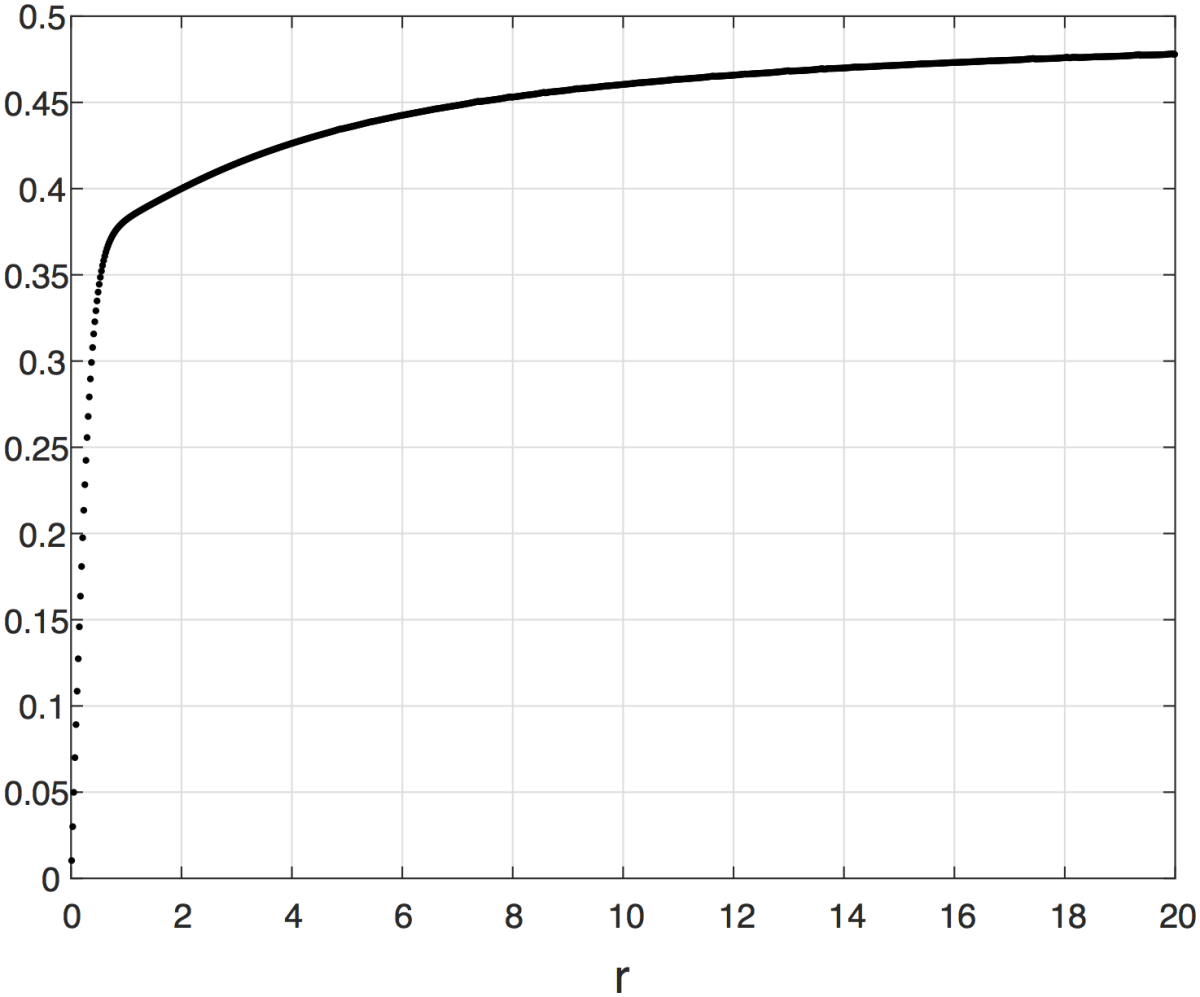
Steady-state output rate *R* as a function of *r* ∈ [0.01, 20] for a EFRBM with *n* = 2, *λ*_*i*_ = 1 for all *i*, and *q* = 1/*r*. Note that the value for *r* = 1 is the steady-state output rate in the RFM.

**Example 4** Consider a EFRBM with *n* = 2 and rates *λ*_0_ = *λ*_1_ = *λ*_2_ = 1. Fig 5 depicts the steady-state output rate *R* as a function of *r*. It may be seen that *R* monotonically increases with *r*. In particular, for *r* = 1 (i.e., the RFM) *R* = 0.3820, wheres for *r* = 20, *R* = 0.4778, that is, the steady-state flow is increased by about 25%. When considering the comparison with the RFM, one should bear in mind that the EFRBM corresponds to an RFM with time-varying rates *η*_*i*_(*t*) that may effectively be much higher than the fixed rates *λ*_*i*_. We assume that the energy that is needed to generate these higher rates comes from the additional interaction forces between the particles.

The next example demonstrates that the increase in *R* as *r* increases is because the neighbor-repelling forces lead to an alleviation of traffic jams.

**Example 5** Consider the EFRBM with dimension *n* = 6, *λ*_0_ = 1.0, *λ*_1_ = 1.2, *λ*_2_ = 0.9, *λ*_3_ = 4.0, *λ*_4_ = 0.2, *λ*_5_ = 1.0, and *λ*_6_ = 1.1. Consider first the case *r* = *q* = 1 (i.e., the RFM). The steady-state density is:

$$\begin{array}{c} e={\left[\begin{array}{cccccc} 0.8443 & 0.8463 & 0.7956 & 0.9510 & 0.1814 & 0.1416 \end{array}\right]}^{\prime }, \end{array}$$

and the corresponding steady-state flow is *R* = 0.1557. Note that since *λ*_3_ is high and *λ*_4_ is low, *e*_1_, *e*_2_, *e*_3_, *e*_4_ ≫ *e*_5_, *e*_6_, indicating a traffic jam at site 4. Consider now the case *r* = 5 (i.e. *q* = 1/5). The steady-state density is now

$$\begin{array}{c} \tilde{e}={\left[\begin{array}{cccccc} 0.5512 & 0.4645 & 0.2549 & 0.8969 & 0.0765 & 0.1963 \end{array}\right]}^{\prime }, \end{array}$$

and the corresponding steady-state output is $\tilde{R}=0.2820$. Note that now the density at site 4 decreased relative to the *r* = 1 case, and that $\tilde{R}>R$. Note also that $\sum_{i=1}^{6}e_{i}=3.7602>\sum_{i=1}^{6}{\tilde{e}}_{i}=2.4403$. This means that the introduction of a “neighbor-repelling” force (i.e. *r* > 1) alleviated the traffic jam, reduced the total steady-state occupancy, and increased the steady-state flow.

**Fig 6:**
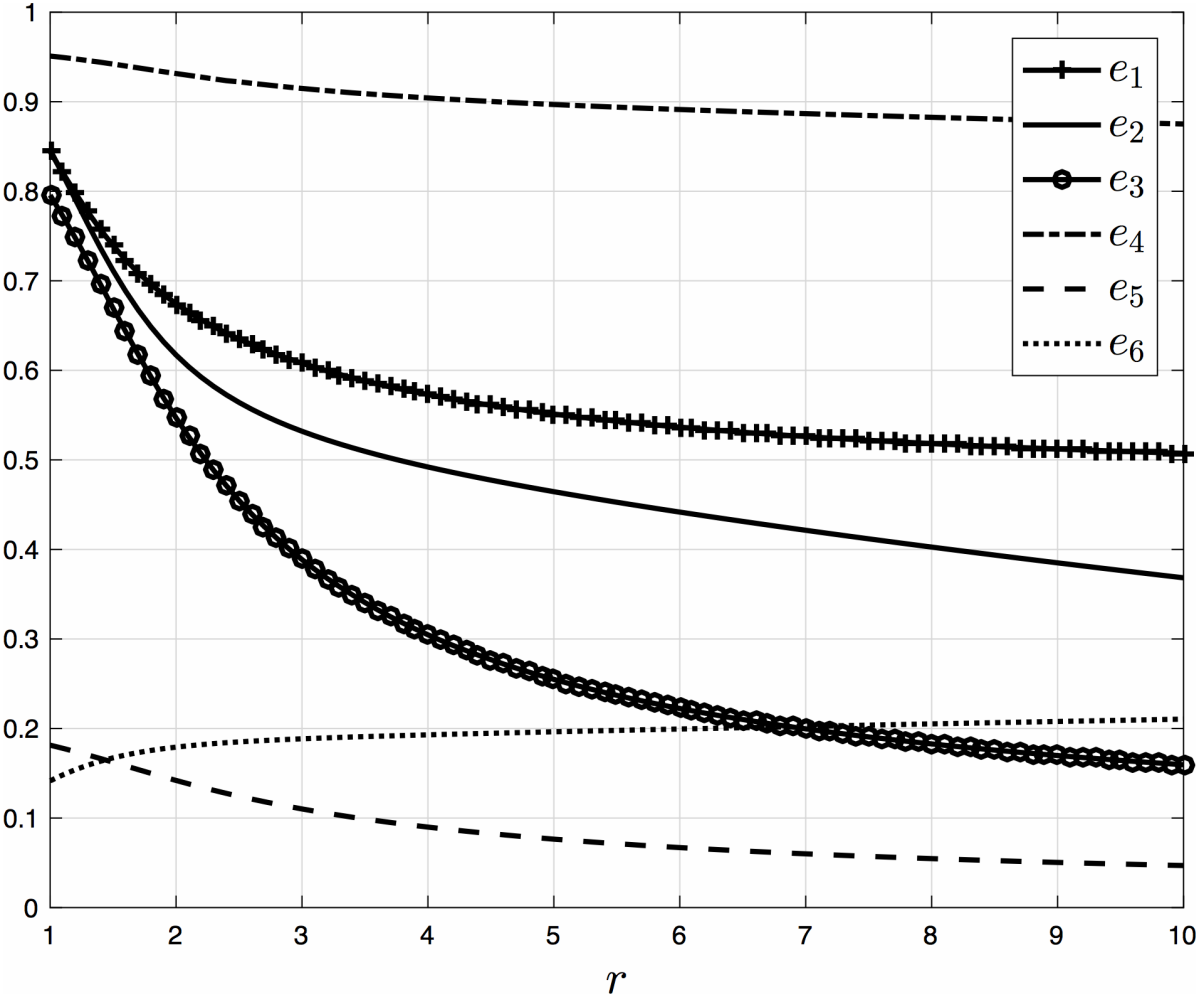
Steady-state densities *e*_*i*_ as a function of *r* ∈ [1, 10] for a EFRBM with *n* = 6, *λ*_0_ = 1.0, *λ*_1_ = 1.2, *λ*_2_ = 0.9, *λ*_3_ = 4.0, *λ*_4_ = 0.2, *λ*_5_ = 1.0, *λ*_6_ = 1.1, and *q* = 1/*r*. Note that as *r* increases all densities become much smaller than one, that is, there are no traffic jams.

Fig 6 depicts the steady-state densities in this example as a function of *r* ∈ [1, 10]. It may be observed that *e*_*i*_, *i* = 1, …, 5, monotonically decreases with *r*, and that *e*_6_ slightly increases with *r*. Note that since the occupancy at site 6 is not affected by *q*, but only by *r*, increasing *r* should indeed increase *e*_6_.

### Extreme interactions

To gain more insight on the effect of the nearest-neighbor interactions on the steady-state behavior, it is useful to consider the cases when *r* → 0 (so $q=\frac{1}{r}\to \infty$) and *r* → ∞ (so $q=\frac{1}{r}\to 0$).

#### The case *r* → 0

Intuitively speaking, a low value of *r* corresponds to: (1) a strong attachment between existing nearest neighbors (small *r*); and (2) a high tendency for moving forward if this involves creating new neighbors (large *q*). As we will see this leads to the formation of traffic jams and, consequently, to a sharp decrease in the output rate.

**Example 6** Consider a EFRBM with dimension *n* = 6 and rates *λ*_*i*_ = 1, *i* = 0, …, 6. For *r* = 0.1 (recall that *q* = 1/*r*), the steady-state values are:

$$\begin{array}{c} e={\left[\begin{array}{cccccc} 0.9908 & 0.9899 & 0.9062 & 0.8978 & 0.9841 & 0.5678 \end{array}\right]}^{\prime },\;\;R=0.0913. \end{array}$$

For *r* = 0.01, the steady-state values are:

$$\begin{array}{c} e={\left[\begin{array}{cccccc} 0.9998 & 0.9999 & 0.9901 & 0.9900 & 0.9899 & 0.4970 \end{array}\right]}^{\prime },\;\;R=0.0099. \end{array}$$

For *r* = 0.005, the steady-state density values are:

$$\begin{array}{c} e={\left[\begin{array}{cccccc} 0.9996 & 0.9999 & 0.9950 & 0.9950 & 0.9950 & 0.4986 \end{array}\right]}^{\prime },\;\;R=0.0050. \end{array}$$

**Fig 7:**
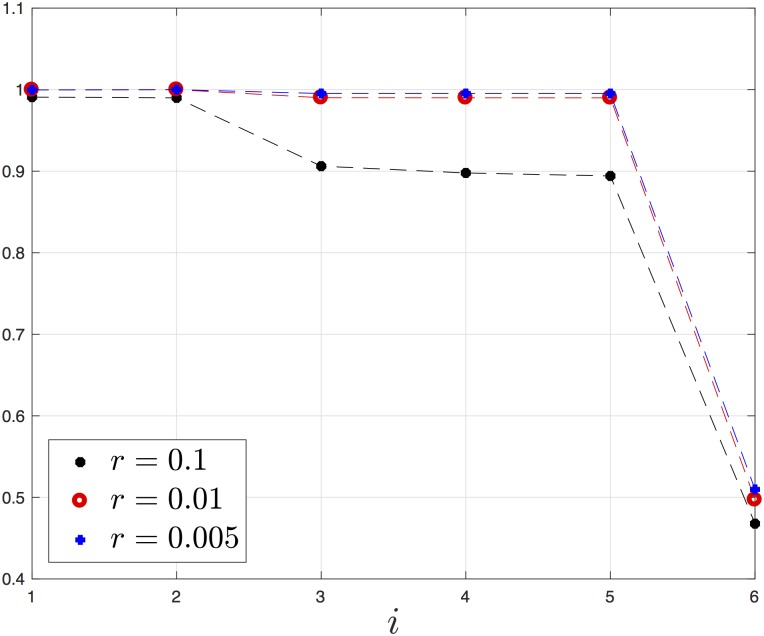
Steady-state densities *e*_*i*_ as a function of *i* for a EFRBM with *n* = 6, *λ*_*i*_ = 1, *i* = 0, …, 6, for three values of *r* (with *q* = 1/*r*).

Fig 7 depicts the steady-state values for the three *r* values. It may be observed that as *r* decreases the density in the first five sites increases to one, i.e. these sites become completely full, and the output rate goes to zero. Note that this highlights the negative effect of traffic jams on the output rate.

We now rigorously analyze the case *r* → 0 for the EFRBM with *n* = 2 and *n* = 3.

**Example 7** Consider the EFRBM with *n* = 2 and *q* = 1/*r*. Expanding *e*_2_ and *e*_1_ in Eq (18) as a Taylor series in *r* yields

$$\begin{array}{c} e_{2}=1-\frac{\lambda_{2}}{\lambda_{1}}r+o\left(r\right),\;e_{1}=1+o\left(r\right), \end{array}$$ (19)

where every *o*(*r*) denotes a function *f*(*r*) satisfying $\lim_{r\to 0}\frac{f(r)}{r}=0$.

Thus, *R* = *λ*_1_ *e*_1_(1 − *e*_2_) = *λ*_2_ *r* + *o*(*r*). This implies in particular that

$$\begin{array}{c} \lim_{r\to 0}e_{1}=\lim_{r\to 0}e_{2}=1,\;\lim_{r\to 0}R=0. \end{array}$$

Thus, when *r* → 0, both steady-state densities go to one (Eq (19) implies that *e*_1_ goes to one faster than *e*_2_), that is, the sites become completely full, and consequently the steady-state output rate goes to zero.

The next result analyzes the case *n* = 3.

**Proposition 4** *The steady-state densities in the EFRBM with* *n* = 3 *satisfy*

$$\begin{array}{ccc} e_{1}\left(r\right) & = & 1-\frac{\lambda_{2}\lambda_{3}}{\lambda_{0}\left(\lambda_{2}+\lambda_{3}\right)}r^{2}+\text{o}\left(r^{2}\right),\\ e_{2}\left(r\right) & = & 1-\frac{\lambda_{3}}{\lambda_{1}}r^{2}+\text{o}\left(r^{2}\right),\\ e_{3}\left(r\right) & = & \frac{\lambda_{2}}{\lambda_{2}+\lambda_{3}}+\text{o}\left(r\right), \end{array}$$ (20)

*and*

$$\begin{array}{c} R\left(r\right)=\frac{\lambda_{2}\lambda_{3}}{\lambda_{2}+\lambda_{3}}r+\text{o}\left(r\right). \end{array}$$ (21)

*Note that this implies that*

$$\begin{array}{c} \lim_{r\to 0}e_{1}\left(r\right)=\lim_{r\to 0}e_{2}\left(r\right)=1,\;\text{and}\;\lim_{r\to 0}R\left(r\right)=0, \end{array}$$

so again as *r* → 0 sites at the beginning of the lattice become completely full and consequently the output rate goes to zero.

Summarizing, as *r* goes to 0 the repelling force between existing neighbors is very weak, and the binding force when forming new neighbors is very strong, leading to the formation of traffic jams at the beginning of the lattice. Consequently, the steady-state flow goes to zero.

We now turn to consider the opposite case, that is, *r* → ∞.

#### The case *r* → ∞

A large value of *r* corresponds to: (1) strong repulsion between existing nearest neighbors (large *r*); and (2) a low tendency for moving forward if this involves creating new neighbors (small *q*). As we will see below, this leads to a phenomena that may be regarded as the opposite of traffic jams, that is, a complete “separation of the densities” along the lattice.

**Example 8** Consider the EFRBM with *n* = 6 sites and rates $\lambda ={\left[\begin{array}{ccccccc} 1 & 1.2 & 0.8 & 0.95 & 1.1 & 0.75 & 1.15 \end{array}\right]}^{\prime }.$ For *r* = 1 (recall that *q* = 1/*r*),

$$\begin{array}{c} e={\left[\begin{array}{cccccc} 0.7322 & 0.6950 & 0.5183 & 0.4558 & 0.4657 & 0.2329 \end{array}\right]}^{\prime },\;\;R=0.2678. \end{array}$$

For *r* = 1,000,

$$\begin{array}{c} e={\left[\begin{array}{cccccc} 0.5262 & 0.0015 & 0.2498 & 0.0022 & 0.2007 & 0.0020 \end{array}\right]}^{\prime },\;\;R=0.4729. \end{array}$$

For *r* = 10,000,

$$\begin{array}{c} e={\left[\begin{array}{cccccc} 0.5261 & 0.0001 & 0.2495 & 0.0002 & 0.2001 & 0.0002 \end{array}\right]}^{\prime },\;\;R=0.4734. \end{array}$$

**Fig 8:**
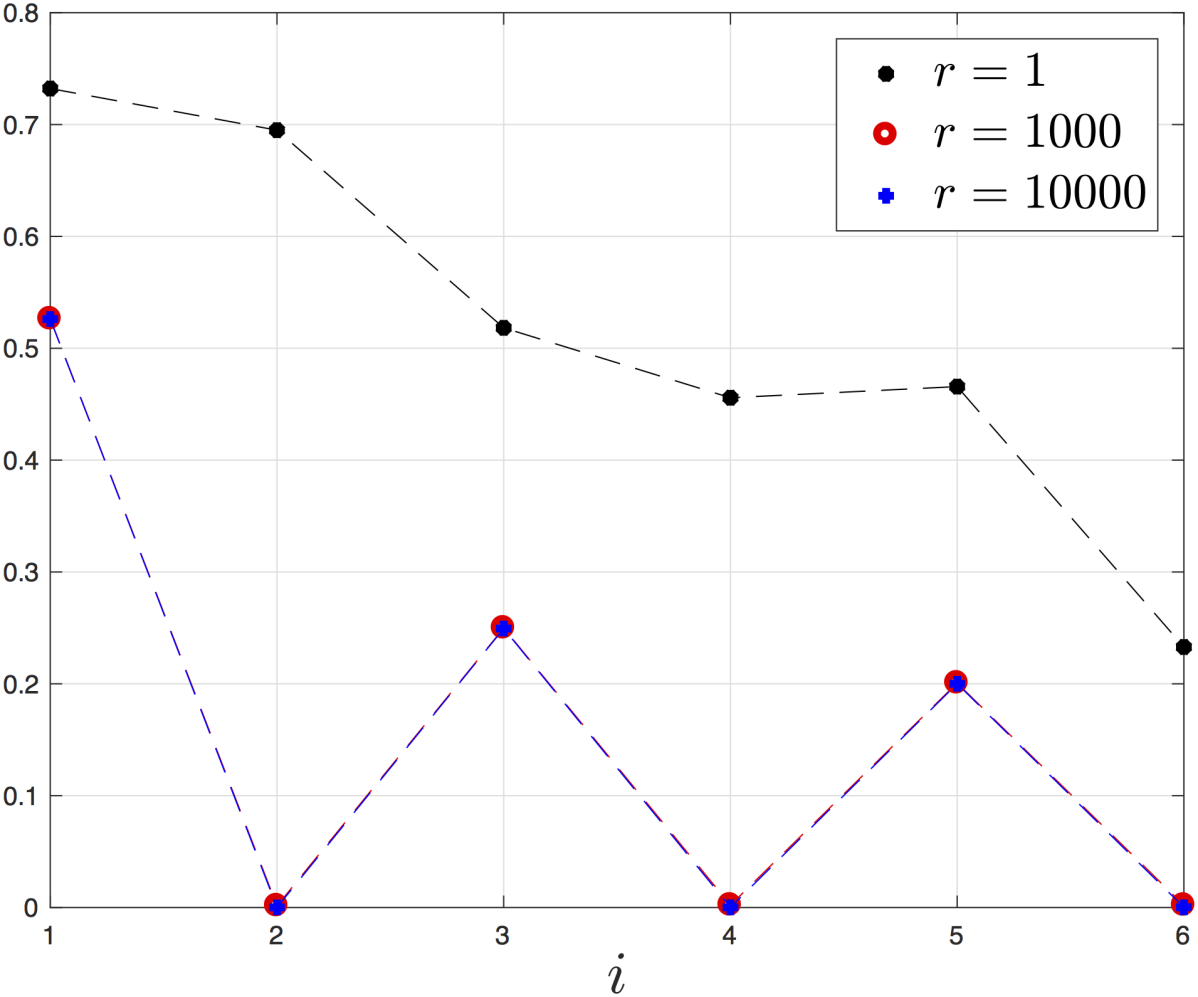
Steady-state densities *e*_*i*_ as a function of *i* for a EFRBM with *n* = 6, *λ*_0_ = 1, *λ*_1_ = 1.2, *λ*_2_ = 0.8, *λ*_3_ = 0.95, *λ*_4_ = 1.1, *λ*_5_ = 0.75, and *λ*_6_ = 1.15, for three values of *r*, and *q* = 1/*r*. The steady-state values for *r* = 1,000 and *r* = 10,000 cannot be distinguished.

Fig 8 depicts these steady-state values for the three *r* values. Note that the steady-state values for *r* = 1,000 and *r* = 10,000 cannot be distinguished. It may be observed that the values *e*_*j*_(*r*), *j* = 2, 4, 6, decrease to zero as *r* increases. In other words, in every pair of consecutive sites one density is very small. This “separation of densities” represents the opposite of a traffic jam. This leads to a substantial increase in the output rate *R* as *r* increases.

We now rigorously analyze the case *r* → ∞ for the EFRBM with *n* = 2 and *n* = 3.

**Example 9** Consider the EFRBM with *n* = 2. Expanding *e* in Eq (18) as a Taylor series in *q* = 1/*r* yields

$$\begin{array}{c} e_{2}=\frac{\lambda_{1}}{\lambda_{2}}q+o\left(q\right),\;e_{1}=\frac{\lambda_{0}}{\lambda_{0}+\lambda_{1}}+o\left(q\right), \end{array}$$

so

$$\begin{array}{c} \lim_{r\to \infty }e_{2}=0,\;\;\lim_{r\to \infty }e_{1}=\frac{\lambda_{0}}{\lambda_{0}+\lambda_{1}},\;\;\lim_{r\to \infty }R=\frac{\lambda_{0}\lambda_{1}}{\lambda_{0}+\lambda_{1}}. \end{array}$$

Thus, in this case the density at site 2 goes to zero, and this yields a positive steady-state output rate.

**Proposition 5** *The steady-state densities in the EFRBM with* *n* = 3 *satisfy*

$$\begin{array}{ccc} e_{1}\left(q\right) & = & a_{1}+b_{1}q+\text{o}\left(q\right),\\ e_{2}\left(q\right) & = & \frac{\lambda_{1}}{\lambda_{2}}q+\text{o}\left(q\right),\\ e_{3}\left(q\right) & = & a_{3}+b_{3}q+\text{o}\left(q\right), \end{array}$$ (22)

*with* *a*_1_, *a*_3_ ∈ (0, 1), *and*

$$\begin{array}{c} R\left(q\right)=\lambda_{0}\left(1-a_{1}\right)+\lambda_{0}\left(\left(a_{1}-1\right)\frac{\lambda_{1}}{\lambda_{2}}-b_{1}\right)q+\text{o}\left(q\right). \end{array}$$ (23)

Note that this implies that

$$\begin{array}{c} \lim_{r\to \infty }e_{2}\left(r\right)=0,\;\text{and}\;\lim_{r\to \infty }R\left(r\right)>0, \end{array}$$

so again as *r* → ∞ the density at site 2 goes to zero and the output rate is positive.

## Discussion

Motor proteins and other moving biological particles interact with their neighbors. Indeed, it is known that cellular cargoes are often moved by *groups* of motor proteins, and recent findings suggest that the bounding time of kinesins on microtubules depend on the presence of neighbors.

To study the effect of such interactions, we introduced a new deterministic compartmental model, the EFRBM, for the flow of particles along an ordered lattice of sites where the transition rates between sites depend both on properties of the lattice and on nearest-neighbor interactions between the particles. The properties of the lattice are modeled using transition rates *λ*_*i*_ between sites. The nearest-neighbor interactions between the particles are modeled using two parameters: *r* that represents the tendency of a moving particle to break from an existing neighbor, and *q* that represents the tendency of a particle to move into a site such that it forms new neighbors (see Fig 2).

The EFRBM is based on a mean-field ansatz neglecting high-order correlations of occupations between neighboring sites. It is possible to use our framework also to derive a more complete model based on binary occupation densities and transitions described by a continuous-time master equation (see, e.g. the interesting paper [44] in which this was done for granular channel transport). However, in such a model the state-variables at time *t* represent the probability of each configuration at time *t*, and the number of possible configurations grows exponentially with the number of sites *n*. On the other-hand, the EFRBM includes *n* (nonlinear) ODEs for *n* sites. Another important advantage of the EFRBM is that it is amenable to analysis using tools from systems and control theory, even in the non-homogeneous case. This allows to rigorously study, for example, the effect of the nearest-neighbors interactions on the steady-state behavior of the EFRBM for any set of transition rates. Our results show that suitable forces between nearby particles can greatly increase the output rate, and reveal that the underlying mechanism for this is the alleviation of traffic jams along the lattice. In particular, when the parameter *r* is very large and *q* is very small, the steady-state density is such that any second site is empty. This represents the “opposite” of a traffic jam, and increases the steady-state flow.

The phenomenological model introduced here may prove useful for other applications as well. For example, an important problem in vehicular traffic is to understand how human drivers react to nearby cars. One may also consider implementing appropriate nearest-neighbor dynamics in algorithms that control autonomous vehicles in order to reduce traffic jams and increase the flow. Of course, implementing this with a very large *r* (or *q*) means very high effective transition rates, but our results suggest that even for *r* not much larger than one the increase in the flow is non-negligible. Another interesting topic for further research is generalizing the EFRBM to include the possibility of attachment/detachment of particles from intermediate sites in the lattice (see [45] for some related ideas).

## Appendix: Proofs

*Proof of Proposition 1.* The fact that *C*^*n*^ is an invariant set of the dynamics follows immediately from the equations of the EFRBM. Let

$$\begin{array}{c} \eta_{i}\left(t\right)≔\lambda_{i}\left(1+\left(q-1\right)z_{i+2}\left(t\right)\right)\left(1+\left(r-1\right)z_{i-1}\left(t\right)\right),\;i=0,\ldots ,n, \end{array}$$ (24)

with the *z*_*i*_s defined in Eq (2). By Eq (3), the EFRBM can be written as

$$\begin{array}{c} {\dot{x}}_{i}\left(t\right)=\eta_{i-1}\left(t\right)x_{i-1}\left(t\right)\left(1-x_{i}\left(t\right)\right)-\eta_{i}\left(t\right)x_{i}\left(t\right)\left(1-x_{i+1}\left(t\right)\right). \end{array}$$ (25)

This is just the RFM (see Eq (1)), but with time-varying rates *η*_*i*_(*t*). Let *a*_*i*_ ≔ min{1, *q*} min{1, *r*}*λ*_*i*_, and *b*_*i*_ ≔ max{1, *q*} max{1, *r*}*λ*_*i*_. It follows from Eq (24) that *a*_*i*_ ≤ *η*_*i*_(*t*)≤*b*_*i*_ for all *i* and for all *t* ≥ 0. Note that for *r*, *q* > 0 every *a*_*i*_ is strictly positive. In other words, all the time-varying rates are uniformly separated from zero and uniformly bounded. Now the proof of Proposition 1 follows from the results in [28].

*Proof of Proposition 2.* Combining the representation in Eq (25) with the uniform boundedness of the rates, Proposition 1, and the results in [43] imply that the EFRBM is *contractive after a small overshoot and short transient* (SOST) on *C*^*n*^. Also, Proposition 4 in [43] implies that for the EFRBM the properties of SOST and SO are equivalent, and this completes the proof.

*Proof of Fact 2.* Consider the EFRBM with *n* = 2 and *q* = 1/*r*. Then Eq (15) becomes

$$\begin{array}{ccc} \lambda_{0}\left(1-e_{1}\right)\left(1+\left(\frac{1}{r}-1\right)e_{2}\right) & = & \lambda_{1}e_{1}\left(1-e_{2}\right)\\ & = & \lambda_{2}e_{2}\left(1+\left(r-1\right)e_{1}\right). \end{array}$$

This yields

$$\begin{array}{c} e_{1}=\frac{\lambda_{2}e_{2}}{\lambda_{1}+\left(\lambda_{2}\left(1-r\right)-\lambda_{1}\right)e_{2}} \end{array}$$ (26)

and

$$\begin{array}{c} a_{1}e_{2}^{2}+a_{2}e_{2}+\lambda_{1}=0, \end{array}$$

with *a*_1_ defined in Eq (17) and *a*_2_ ≔ −*λ*_1_ − *λ*_2_ − *a*_1_. The feasible solution (i.e. the one satisfying *e*_1_, *e*_2_ ∈ (0, 1) for any set of parameter values) is given by

$$\begin{array}{c} e_{2}=\frac{-a_{2}-\sqrt{a_{2}^{2}-4a_{1}\lambda_{1}}}{2a_{1}}, \end{array}$$

and Eq (26).

*Proof of Prop. 3.* To emphasize the dependence on the parameters, write the EFRBM as $\dot{x}=f\left(x;v\right)$, where $v≔{\left[\begin{array}{ccccc} \lambda_{0} & \ldots & \lambda_{n} & r & q \end{array}\right]}^{\prime }$. Note that *f* is an analytic function. Then the steady-state satisfies the relation *f*(*e*;*v*) = 0. The Jacobian matrix of this relation with respect to *x* is

$$\begin{array}{c} J\left(x;v\right)≔\frac{\partial }{\partial x}f\left(x;v\right), \end{array}$$

which is just the Jacobian of the dynamics. Fix $v^{0}\in R_{++}^{n+3}$ and let *e*^0^ ∈ Int(*C*^*n*^) denote the corresponding steady-state, that is, *f*(*e*^0^; *v*^0^) = 0 and *e*^0^ = *h*(*v*^0^). Suppose that there exists a matrix measure $\mu :R^{n\times n}\to R$ such that *μ*(*J*(*e*^0^; *v*^0^)) < 0. This implies in particular that *J*(*e*^0^, *v*^0^) is Hurwitz (see e.g. [46]), so it is not singular and invoking the implicit function theorem implies that the mapping *h* is analytic. It follows from the results in [28] that such a matrix measure *μ* indeed exists, and this completes the proof.

*Proof of Prop. 4.* Expand *e*_*i*_, *i* = 1, 2, 3, as

$$\begin{array}{c} e_{i}=a_{i}+b_{i}r+c_{i}r^{2}+\text{o}\left(r^{2}\right). \end{array}$$ (27)

Recall that the steady-state equations are given by *R*(*e*) = *g*_0_(*e*) = *g*_1_(*e*) = … = *g*_3_(*e*), with the *g*_*i*_s given in Eq (4). Substituting Eq (27) yields

$$\begin{array}{ccc} g_{0}\left(e\right) & = & \frac{\lambda_{0}\left(1-a_{1}\right)a_{2}}{r}+\ldots ,\\ g_{1}\left(e\right) & = & \frac{\lambda_{1}\left(1-a_{2}\right)a_{3}}{r}+\ldots ,\\ g_{2}\left(e\right) & = & \lambda_{2}\left(a_{1}-1\right)a_{2}\left(a_{3}-1\right)+\ldots ,\\ g_{3}\left(e\right) & = & \lambda_{3}\left(1-a_{2}\right)a_{3}+\ldots . \end{array}$$

Since *R*(*e*) is bounded, we conclude that

$$\begin{array}{c} \left(1-a_{1}\right)a_{2}=\left(1-a_{2}\right)a_{3}=0. \end{array}$$ (28)

Assume for the moment that *a*_2_ = 0. Then

$$\begin{array}{ccc} g_{2}\left(e\right) & = & \lambda_{2}\left(a_{1}-1\right)\left(a_{3}-1\right)b_{2}r+\ldots ,\\ g_{3}\left(e\right) & = & \lambda_{3}a_{3}+\ldots , \end{array}$$ (29)

and this implies that *a*_3_ = 0. Now, *g*_1_(*e*) = *λ*_1_(1 + *b*_3_) + … and combining this with Eq (29) yields *b*_3_ = −1. Thus, *e*_3_ = *a*_3_ + *b*_3_ *r* + *c*_3_ *r*^2^ + o(*r*^2^) = −*r* + o(*r*), and this is a contradiction as *e*_3_(*r*) will be strictly negative for any *r* > 0 sufficiently small. We conclude that *a*_2_ ≠ 0, so Eq (28) yields *a*_1_ = 1, and also (1 − *a*_2_)*a*_3_ = 0. Suppose that *a*_3_ = 0. Then

$$\begin{array}{ccc} g_{0}\left(e\right) & = & -\lambda_{0}a_{2}b_{1}+\ldots ,\\ g_{1}\left(e\right) & = & \lambda_{1}\left(1+b_{3}\right)\left(1-a_{2}\right)+\ldots ,\\ g_{2}\left(e\right) & = & \lambda_{2}a_{2}\left(b_{1}-1\right)r+\ldots ,\\ g_{3}\left(e\right) & = & \lambda_{3}\left(1-a_{2}\right)b_{3}r+\ldots . \end{array}$$

It follows that *a*_2_ *b*_1_ = (1 + *b*_3_)(1 − *a*_2_) = 0. Since we already know that *a*_2_ ≠ 0, *b*_1_ = 0. The case *b*_3_ = −1 is impossible, as then *e*_3_(*r*) < 0 for *r* > 0 sufficiently small, so *a*_2_ = 1. But then *R*(*e*) = *g*_2_(*e*) = −*λ*_2_ *r* + … and this is a contradiction. We conclude that *a*_3_ ≠ 0, so Eq (28) yields *a*_2_ = 1. Summarizing, we have *a*_1_ = *a*_2_ = 1. Now,

$$\begin{array}{ccc} g_{0}\left(e\right) & = & -\lambda_{0}b_{1}+\ldots ,\\ g_{1}\left(e\right) & = & -\lambda_{1}b_{2}a_{3}+\ldots ,\\ g_{2}\left(e\right) & = & \lambda_{2}\left(a_{3}-1\right)\left(b_{1}-1\right)r+\ldots ,\\ g_{3}\left(e\right) & = & \lambda_{3}\left(1-b_{2}\right)a_{3}r+\ldots . \end{array}$$

This gives *b*_1_ = 0 and *b*_2_ *a*_3_ = 0. Since we already know that *a*_3_ ≠ 0, *b*_2_ = 0. Now,

$$\begin{array}{ccc} g_{0}\left(e\right) & = & -\lambda_{0}c_{1}r+\ldots ,\\ g_{1}\left(e\right) & = & -\lambda_{1}a_{3}c_{2}r+\ldots ,\\ g_{2}\left(e\right) & = & \lambda_{2}\left(1-a_{3}\right)r+\ldots ,\\ g_{3}\left(e\right) & = & \lambda_{3}a_{3}r+\ldots . \end{array}$$

Equating the coefficients here yields $a_{3}=\frac{\lambda_{2}}{\lambda_{2}+\lambda_{3}}$, $c_{1}=\frac{-\lambda_{2}\lambda_{3}}{\lambda_{0}\left(\lambda_{2}+\lambda_{3}\right)}$, and *c*_2_ = −*λ*_3_/*λ*_1_. Since we know that the steady-state equations admit a unique solution this yields Eq (20), and the equation *R*(*e*) = *g*_0_(*e*) yields Eq (21).

*Proof of Prop. 5.* Expand *e*_*i*_, *i* = 1, 2, 3, as

$$\begin{array}{c} e_{i}=a_{i}+b_{i}q+c_{i}q^{2}+\text{o}\left(q^{2}\right). \end{array}$$ (30)

Recall that the steady-state equations are given by *R*(*e*) = *g*_0_(*e*) = *g*_1_(*e*) = … = *g*_3_(*e*), with the *g*_*i*_s given in Eq (4). Substituting Eq (30) yields

$$\begin{array}{ccc} g_{0}\left(e\right) & = & \lambda_{0}\left(a_{1}-1\right)\left(a_{2}-1\right)+\ldots ,\\ g_{1}\left(e\right) & = & \lambda_{1}a_{1}\left(a_{2}-1\right)\left(a_{3}-1\right)+\ldots ,\\ g_{2}\left(e\right) & = & \lambda_{2}\frac{a_{1}a_{2}\left(1-a_{3}\right)}{q}+\ldots ,\\ g_{3}\left(e\right) & = & \lambda_{3}\frac{a_{2}a_{3}}{q}+\ldots . \end{array}$$

This implies that

$$\begin{array}{c} a_{1}a_{2}\left(1-a_{3}\right)=a_{2}a_{3}=0. \end{array}$$ (31)

Assume for the moment that *a*_2_ ≠ 0. Then *a*_3_ = *a*_1_ = 0. This yields

$$\begin{array}{ccc} g_{0}\left(e\right) & = & \lambda_{0}\left(1-a_{2}\right)+\ldots ,\\ g_{1}\left(e\right) & = & \lambda_{1}\left(1-a_{2}\right)b_{1}q+\ldots ,\\ g_{2}\left(e\right) & = & \lambda_{2}a_{2}\left(1+b_{1}\right)+\ldots . \end{array}$$

This implies that *a*_2_ = 1 and *b*_1_ = −1. This yields *e*_1_(*r*) = −*r* +o(*r*) which is a contradiction.

We conclude that *a*_2_ = 0. Now,

$$\begin{array}{ccc} g_{0}\left(e\right) & = & \lambda_{0}\left(1-a_{1}\right)+\ldots ,\\ g_{1}\left(e\right) & = & \lambda_{1}a_{1}\left(1-a_{3}\right)+\ldots ,\\ g_{2}\left(e\right) & = & \lambda_{2}a_{1}\left(1-a_{3}\right)b_{2}+\ldots ,\\ g_{3}\left(e\right) & = & \lambda_{3}a_{3}\left(1+b_{2}\right)+\ldots . \end{array}$$

Equating the coefficients here yields the following. First, *a*_1_ ≠ 0, and since *e*_1_(*q*) = *a*_1_ + …, this implies that *a*_1_ > 0. Second, if *a*_3_ = 1 then *a*_1_ = 1 and *b*_2_ = −1 which is a contradiction as then *e*_2_(*q*) = −*q* + *o*(*q*). Thus, *a*_3_ ≠ 1 and this implies that *a*_1_ ≠ 1. We conclude that *a*_1_, *a*_3_ ∈ (0, 1). Now the equations for *g*_1_ and *g*_2_ yield *b*_2_ = *λ*_1_/*λ*_2_. This proves Eq (22). Expanding *g*_0_ up to order one in *q*, and using *R* = *g*_0_ yields Eq (23).
